# The protective effect of bone marrow-derived mesenchymal stem cells in liver ischemia/reperfusion injury via down-regulation of miR-370

**DOI:** 10.22038/ijbms.2019.32670.7812

**Published:** 2019-06

**Authors:** Mohammad Ali Zare, Abdolhossein Zare, Negar Azarpira, Sara Pakbaz

**Affiliations:** 1 Transplant Research Center, Shiraz University of Medical Sciences, Shiraz, Iran; 2 Department of Pathology, University of Toronto, Toronto, Canada

**Keywords:** Apoptosis, Bcl2, BAX, Ischemia reperfusion injury, Mesenchymal stem cells, microRNA 370

## Abstract

**Objective(s):**

Liver transplantation is the most important therapy for end-stage liver disease and ischemia reperfusion (I/R) injury is indeed a risk factor for hepatic failure after grafting. The role of miRNAs in I/R is not completely understood. The aim of this study was to investigate the potential protective role of the mesenchymal stem cells (MSCs) and ischemic preconditioning on miR-370 expression and tissue injury in hepatic I/R injury.

**Materials and Methods:**

In this study, 24 BALB/c mice were divided into 4 groups, including sham, I/R, I/R mouse that received MSCs (I/R+MSC) and ischemia preconditioning (IPC) The expression levels of hepatic miR-370, Bcl2 and BAX in male BALB/c mice in different groups including hepatic I/R, hepatic I/R received MSCs, and hepatic I/R with IPC were assessed by quantitative real-time PCR. The effect of miR-370 on hepatic I/R was investigated by serum liver enzyme analysis and histological examination.

**Results:**

The expression of miR-370 was significantly up-regulated in the mice subjected to hepatic I/R injury as compared with the sham operated mice. Injection of MSCs led to the down-regulation of the serum liver enzymes, expression of miR-370 and BAX, up-regulation of Bcl2 as well as the improvement of hepatic histological damage. IPC led to similar results, but the difference was not significant.

**Conclusion:**

Our data suggest that miR-370 affected the Blc2/BAX pathway in hepatic I/R injury, and down- regulation of miR-370 by BM-MSCs efficiently attenuated the liver damage.

## Introduction

Hepatic ischemia/reperfusion (I/R) injury is responsible for hepatocellular damage during clinical procedures such as trauma, hepatic artery ligation, and liver transplantation (1). Hepatic transplantation is regarded as a life-saving treatment for end-stage liver diseases. However, I/R is an inevitable event and usually leads to both delay or even loss of transplanted organ and higher incidence of acute and chronic rejection (2). Ischemic stress involves nutrient deprivation, hypoxia, acidosis, and altered levels of various ions and metabolites. Reperfusion, which abruptly alters these parameters, is a second stress to already stressed cells. The major mechanisms of I/R are mainly derived from cellular deprivation from oxygen and nutrients, with abrupt exposure to a second stress, generation of toxic reactive oxygen species (ROS) after blood reperfusion of ischemic tissues (3, 4).

Fundamental features of I/R are ROS release, damage and activation of endothelial cells, emigration of neutrophils, release of cytokines/chemokines with subsequent activation of apoptosis and parenchymal damage. The current therapeutic strategies, such as pharmacological and surgical interventions like ischemic preconditioning are applied in order to decrees this damage (5).

In ischemic preconditioning, a brief ischemic time period followed by organ reperfusion elicited a protective effect on I/R.

Mesenchymal stem cells (MSCs) are multipotent stromal cells with the capability of self-renewal and differentiation into a variety of cells (6).These cells display immune-modulatory properties with paracrine mechanisms which is important in suppression of tissue inflammation during active phase of I/R (7). In renal I/R model in rats, using microvesicles derived from human Wharton’s Jelly MSCs ameliorates tissue damage by suppressing CX3CL1, alleviating renal cell apoptosis and decreasing tissue inflammation and CD68+ macrophages. MSCs have an immune-modulatory activity with suppression of inflammation during I/R. Microvesicles are membranous structures derived from MSCs that contain bioactive molecules, such as proteins, mRNAs and micro-RNAs (8, 9). By using MSCs derived microvesicles in renal I/R injury , VEGF and proangiogenetic factors were up-regulated and renal fibrosis was decreased in long term (10). In liver I/R, MSCs derived exosomes markedly suppressed the histological and biochemical parameters in the liver I/R in rats. The hepatocyte apoptosis and tissue inflammation were significantly decreased. Pan and colleagues, demonstrated that inhibition of MEK/ERK pathway played an important role in this protection (11).

MicroRNAs (miRNAs) are small non-coding single stranded RNAs that interact with the 3’ untranslated region (3’-UTR) of their targets with subsequent mRNA cleavage or translational repression. Li and colleagues reported that miR-370 expression was significantly up-regulated during hepatic I/R, and its inhibition attenuated the liver damage (12).

The protective effects of MSCs and IPC on liver I/R has been previously mentioned. We supposed that in this process, a paracrine mechanism of MSCs on miRNA expression may be responsible for its protective effect. In this study, we investigated the potential effect of intraportal injection of BM-MSCs and IPC on miR-370 gene expression level in mouse I/R model.

## Materials and Methods

***Animal model and study groups***

Male BALB/c mice, weighing about 20-25 g and aged 6-8 weeks were used. The mice were housed under standard conditions with a 12 hr light-dark cycle at the Animal Center of Shiraz University of Medical Sciences. Food and water were freely available. All the experimental procedures were approved by the Animal Care Committee of our university. Twenty-four BALB/c mice were divided into four groups of sham, I/R, I/R mouse that received MSCs (I/R+MSC) and IPC. Mice were intraportally injected with 1×10^6^ BM-MSCs labeled with Fluorescent dye 1,1′-dioctadecyl-3,3,3′,3′- tetramethylindodicarbocyanine, 4-chlorobenzenesulfonate salt (DID). The sham group mice were injected with 1 ml of phosphate-buffered saline (PBS). All mice were sacrificed after 6 and 24 hr reperfusion and their livers were dissected for histopathological and migration analysis.

***Bone marrow-derived mesenchymal stem cells (BM-MSCs) isolation, culture and characterization***

BM-MSCs were isolated from donor mice. Animals were sacrificed and both femur bones were removed. The femur bones were cut on both ends and each core was flushed with culture medium. The bone marrow tissue was obtained and seeded in culture flasks (Nunc, Germany) and cultured under standard conditions using DMEM (Dulbecco’s Modified Eagle Medium) containing 10% fetal bovine serum (FBS) supplemented with 100 U/ml penicillin, 100 µg/ml streptomycin (GIBCO, USA). MSCs between the fourth and fifth passages were used for the mentioned experiments. The cells were stained with antibodies against mouse antigens CD44, CD34, Sca-1, CD45 and compared with corresponding isotype control using flow cytometry (BD Pharmingen, BD FACSCalibur, United States). The osteogenic and adipogenic potential was also evaluated.

***Hepatic I/R model***

A nonlethal model of partial (70%) hepatic ischemia was achieved as previously described (13). The mice were anesthetized with intraperitoneal injection of ketamine (100 mg/kg) and xylazine ( 10 mg/kg). The abdomen was opened through a midline incision. The branches of the portal vein, hepatic artery, and the bile duct which were provided with the median and the left lateral lobes of the liver were clamped using a microvessel clip for 60 min to induce partial hepatic ischemia. Then, the vessel clip was removed and livers were reperfused for 6 and 24 hr. Immediately after reperfusion, 1×10^6 ^ MSCs suspended in 200 μl of PBS were injected into the I/R-MSCs group via the portal vein (n=6). The same volume of PBS was transfused into the I/R group (n=6) mice. The sham group (n=6) underwent the same operation.

The IPC (n=6) group underwent 10 min of hepatic ischemia and 10 min of reperfusion prior to the subsequent prolonged ischemic insult.

***Tracking of DID-labeled MSCs in the experimental mice***

To detect the presence of the injected DID -labeled MSCs in the experimental animals, we followed the location of the floursent labeled cells, using *in vivo* imaging instrument (Kodak, United States).

***Histopathology findings***

Tissue samples from the left lateral hepatic lobe were fixed in 10% neutral-buffered formalin, embedded in paraffin, sectioned at 4 μm thickness, and stained with hematoxylin and eosin (H&E). Subsequently, the results were confirmed by a pathologist. Tissue slides were evaluated by a pathologist who was blinded as to the examined groups. Liver damage was evaluated using the Suzuki score (14). The histopathologic findings of congestion, vacuolization and necrosis were evaluated and total score was recorded from 0-16.

***Biochemical assays***

Whole blood samples were obtained by sterile cardiac puncture and serum was collected. Serum aspartate aminotransferase (AST) and alanine aminotransferase (ALT) levels were measured as markers of hepatocyte injury, using an Auto Analyzer RA1000 (TECHNICON, United States).

**Figure 1:**
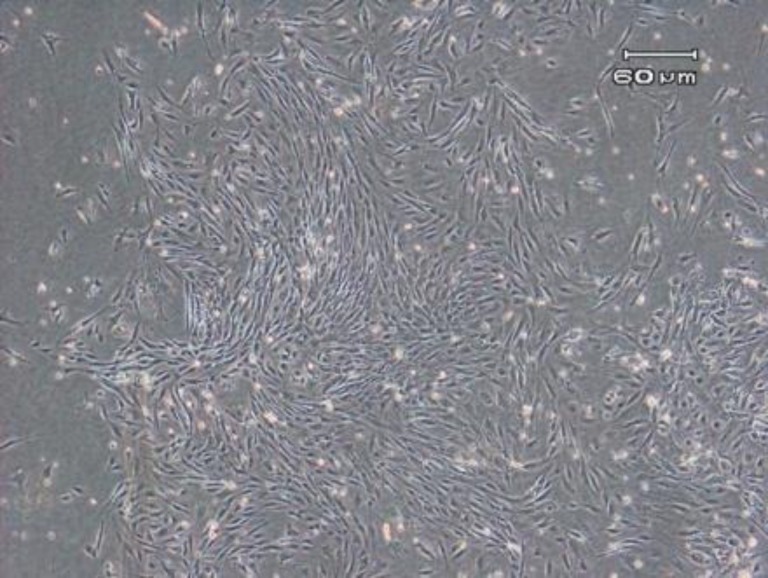
The cytomorphology of BM-MSCs. BM-MSCs from passage 3 displayed a spindle-shape morphology

**Figure 2:**
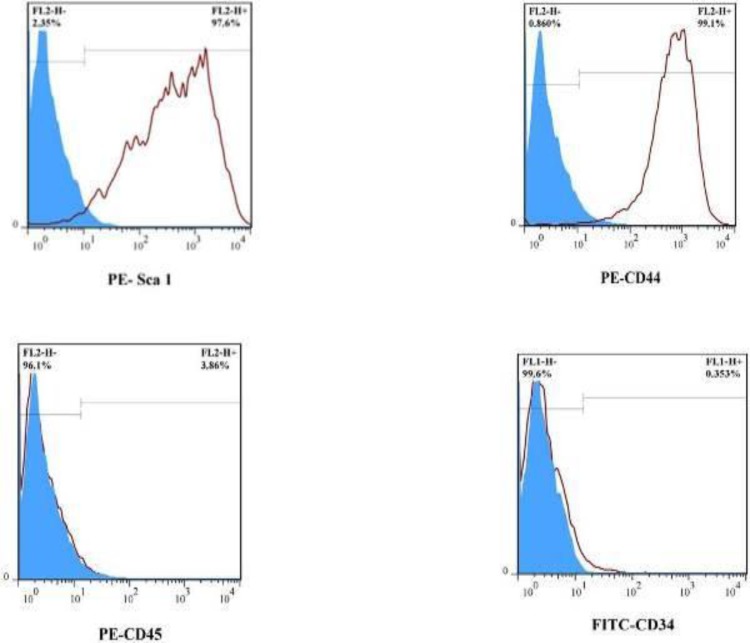
Cell surface markers of BM-MSCs. The results showed that > 97% of cells were positive for CD44 and Sca-1, while < 3% of them were positive for CD45 or CD34

**Figure 3:**
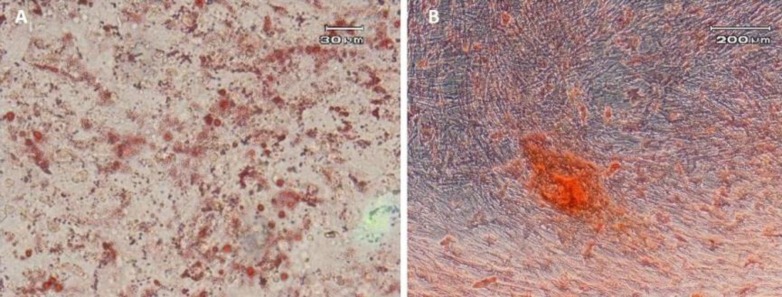
Adipogenic and osteogenic differentiation of BM-MSCs

The figure (A) and (B) show fat droplet and calcium deposition respectively

**Figure 4:**
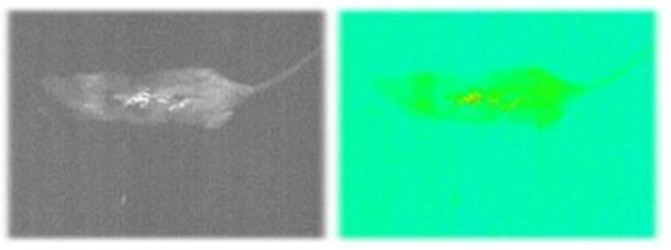
*In vivo* tracking BM-MSCs. The BM-MSCs detected 24 hr after injection in the liver organ

**Figure 5:**
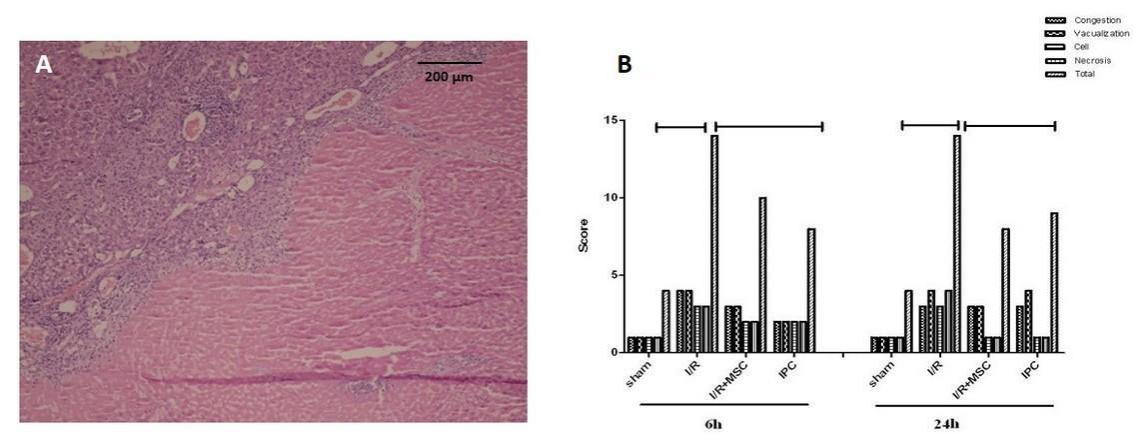
Histopathology of liver tissue (H&E ×200)

(A) congestion, vacuolization, necrosis, and inflammatory cell infiltration were decreased in the I/R+ MSC and IPC groups when compared to I/R group (B) but it was not significant in both 6 hr and 24 hr after reperfusion (Suzuki scores), (*P>*0.05)

**Figure 6:**
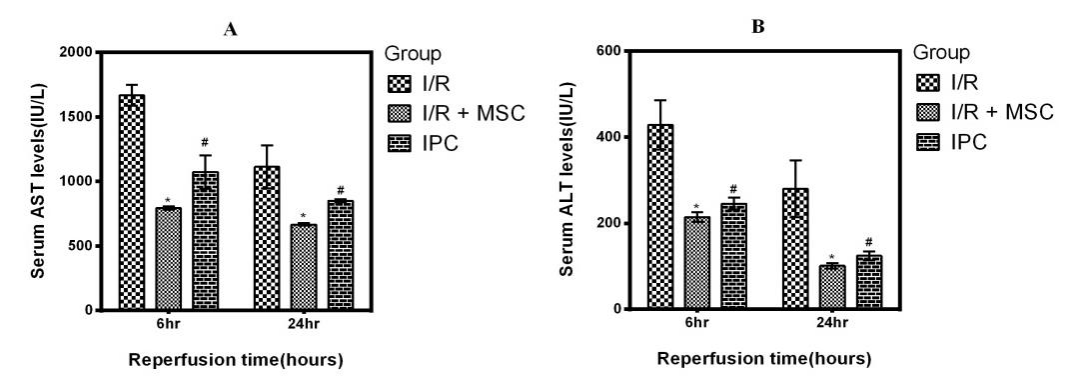
Serum ALT and AST levels following 6 and 24 hr reperfusion

(A) serum AST levels and (B) serum ALT levels. *Significant change in I/R+MSCs compared to I/R group; # Significant change in IPC mouse compared to I/R group

The results are presented from 3 independent experiments

**Figure 7:**
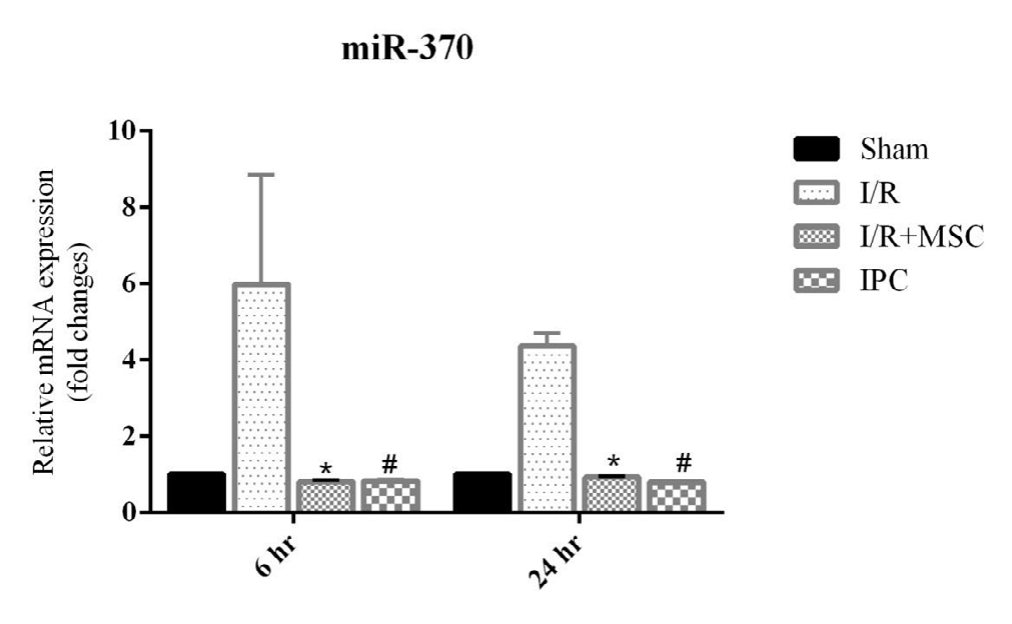
Expression level of miR-370 was increased in the I/R group and significantly decreased in I/R+MSCs and IPC groups after 24 hr reperfusion. The decreased level after 6 hr reperfusion was not significant. *Significant change in I/R+MSCs compared to I/R group. # Significant change in IPC mouse compared to I/R group

The results are presented from 3 independent experiments

**Figure 8:**
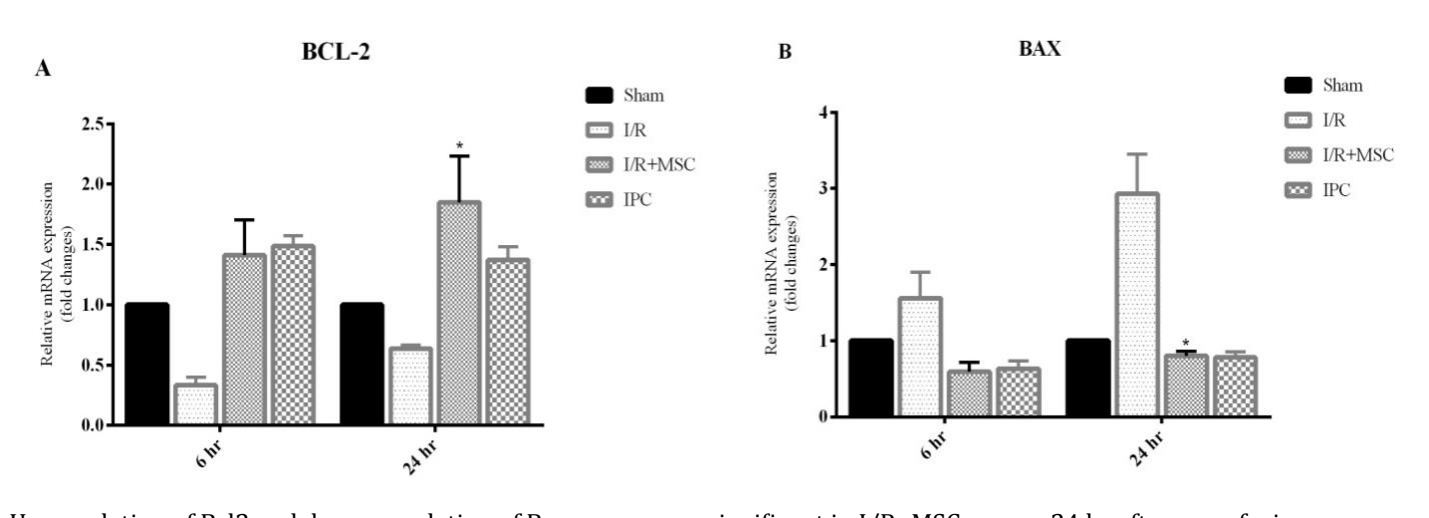
Up-regulation of Bcl2 and down-regulation of Bax genes were significant in I/R+MSCs group 24 hr after reperfusion

(A) Bcl2 gene expression and (B) Bax gene expression. *Significant change in I/R+MSCs compared to I/R group

The results are presented from 3 independent experiments

***Quantitative RT-PCR for miR-370 gene expression***

Total RNA from 50 mg of liver tissue was isolated using TRIzol Reagent (Life Technologies, Rockville, Md, USA) according to the manufacturer’s instructions. The quantity of the extracted RNA was evaluated by Nanodrop (measuring the optical density 260/280), and the quality of RNA was assessed by running 3 μl on 1% agarose gel. The good quality was indicated by the lack of a smear and presence of 28S and 18S ribosomal RNA ( rRNA). Then, cDNA was synthesized using the polyA tail reverse-transcription primer (PARSGENOME MiR-Amp kit, Iran). Small nuclear RNA U6 (U6snRNA) was also considered as the internal control.

***Quantitative RT-PCR for Bcl-2 and Bax gene expression***

Total RNA from liver tissue was reversely transcribed into cDNA using Prime Script RT Reagent Kit (Takara, Japan) according to the manufacturer’s guidelines. Glyceraldehyde 3-phosphate dehydrogenase gene (GAPDH) was used as internal control. The primer sequences used were: 

Bcl-2-F, 5’-GGATAACGGAGGCTGGGATGC-3’; 

Bcl-2-R, 5’- ATTTGTTTGGGGCAGGTTTGTCG-3’;

Bax-F,5’-TTTTGCTACAGGGTTTCATCCAGG-3’;

Bax-R,5’-ATCATCCTCTGCAGCTCCATATTG-3’;

GAPDH-F,5’-ACTGAGCAAGAGAGGCCCTA-3’;

GAPDH-R,5’- TATGGGGGTCTGGGATGGAA-3’. 

The expression level of miR-370, Bcl-2 and Bax was determined by Livak (2^-ΔΔCT^) method. Melt curve was also analyzed to confirm the specificity of reaction at the end of program.

***Statistical analysis***

All experiments were repeated at least three times. The results were expressed as mean±SD values. Significant differences were determined using Kruskal-Wallis for multiple comparisons by SPSS software (SPSS: An IBM Company, version 19.0, IBM Corporation, Armonk, NY, USA). *P*-values less than 0.05 were considered signiﬁcant.

## Results

***Characterization of BM-MSCs***

The plastic-adherent cells from bone marrow formed a monolayer with fibroblast-like morphology (Figure 1). The flow cytometry results demonstrated that the BM-MSCs expressed stromal markers (Sca-1, CD44) and negative for hematopoietic marker (CD34 and CD45) (Figure 2). After differentiation toward adipogenic and osteogenic lineage, the presence of lipid vacuoles was confirmed by Oil Red O, and calcium deposition was revealed with Alizarin Red (Figure 3).

***In vivo tracking of DID labeled BM-MSCs.***

The DID-positive MSCs remained in the liver organ after 24 hr (Figure 4).

***Histopathology of liver***

The histopathological findings such as congestion, hepatocyte vacuolization, necrosis, and inflammatory cell infiltration were decreased in the I/R+MSC and IPC groups when compared to I/R group, but it was not significant during 6 and 24 hr after reperfusion (*P>* 0.05), (Figure 5).

***Liver function***

The serum AST and ALT levels were significantly increased at both 6 and 24 hr following I/R injury. In IPC group and animals that received MSCs, liver enzymes were significantly decreased 6 hr and 24 hr after reperfusion (*P<*0.05); this indicates that both treatments produce similar improvement in liver function (Figure 6).

***Expression of miR-370 gene in liver tissues***

Exposure of mice to I/R leads to a significantly increase in miR-370 gene expression. During 6 hr after reperfusion, miR-370 gene expression was not significantly decreased in both groups. The elevated miR-370 expression levels were significantly decreased 24 hr after reperfusion (*P*<0.05), (Figure 7).

***Expression of Bcl-2 and Bax genes in liver tissues***

Exposure to I/R leads to a significant decrease in the antiapoptotic Bcl2 gene expression and significant increase in the proapoptotic Bax gene expressions, following 24 hr of reperfusion in the liver tissues that received MSCs (*P*<0.05)**.** A similar pattern was observed after 6 hr reperfusion, but the fold change was not significant (Figure 8).

## Introduction

In the present study, MSCs were injected into I/RI mice by the portal vein after I/R injury in order to investigate the effect of MSCs and IPC in I/RI mice. Results showed that after MSCs infusion and IPC treatment serum ALT and AST levels were decreased in the I/R+MSCs and IPC groups compared to I/R group on the 6 and 24 hr after reperfusion. Furthermore, the expression of miR-370 and Bax genes on the both of time in the I/R+MSCs and IPC groups were decreased compared to I/R group, but there was no significant difference in 6 hr after reperfusion. On the other hand, Bcl-2 expression was increased in the I/R+MSCs and IPC groups on the both of time compared to I/R group. The result showed that MSCs was strong influence on I/RI mice than IPC. 

 Recent evidence has shown that miRNAs are involved in various biological processes, such as cell differentiation, organ morphogenesis, oncogenesis, and hematopoietic lineage differentiation (15, 16). MiR-370 is recently discovered and has important functional roles in several human tumors. Target Scan (http://www.targetscan.org/), miRmap (http://mirmap.ezlab.org/), and miRWalk (http://zmf.umm.uni-heidelberg.de/) online programs are utilized to predict the target genes for miR-370. Overexpression of miR-370 in gastric cancer cells promoted the tumor cell proliferation, along with down-regulation of its targets, TGFβ-RII and FOXO1. The same pattern was confirmed in prostatic carcinoma (17). In the other research which was conducted by Sun and colleague, the expression levels of miR-370 were significantly down-regulated in HepG2 liver cancer cells and transfection with miR-370 mimics significantly decreased the proliferation of cells. They concluded that miR-370 worked as a tumor suppressor gene, and promoted cell death by regulating the Akt/FoxO3a signaling pathway (18). This finding was confirmed in laryngeal squamous cell carcinoma (18, 19). These findings are inconsistent with the results in gastric cancers. This controversy was partly due to single miRNA targeting different mRNA in various tumor types.

The significance of miR-370 with TGFβ-RII and FOXO1 signaling pathways was also evaluated in other non-tumor conditions. MiR-370 was up-regulated in hepatic ischemia reperfusion injury, and liver injury was significantly attenuated after its inhibition (20). Also, Li and colleagues revealed that miR-370 was increased after hepatic I/R and its inhibition led to the down-regulation of liver enzymes and proinflammatory cytokines. They confirmed that miR-370 targeted the 3’ untranslated region of transforming growth factor-β receptor II (TβRII) with a potential role by targeting transforming growth factor-β receptor II (12). Iliopoulos and colleagues showed that miR-370 affects lipid metabolism and triglycerides accumulation in the liver cells by targeting carnitine palmitoyl transferase 1 alpha (Cpt1α). The hepatocytes have critical roles in regulation of energy balance by controlling the metabolism of carbohydrates, lipid and proteins.During ischemia, cellular deprivation from oxygen and nutrients as well as generation of toxic ROS during blood reperfusion resulted in significant damage to these metabolic pathways with subsequent hepatocyte damage (21).

Recently, plasma level of miR-370 has been reported as a promising non-invasive biomarker in patients with coronary artery disease, in which cardiomyocyte ischemia had occurred. This molecule participates in homeostasis and lipid metabolism of cardiac muscles (22). In myocardial infarction or acute ischemia/reperfusion, cardiomyocytes are vulnerable to oxidative stress-induced injury. FOXO1 had a significant anti-apoptotic effect against oxidative stress in cardiomyocytes (23)**.**

The forkhead-box class O (FOXO) transcription factors has an important role in the IGF-1/Pl3K/Akt signal transduction pathway. This transcription factor is considered as a critical mediator of oxidative stress resistance by cross talk between apoptosis and autophagy. Depending on the cell type and severity of injury, FOXO transcription factors regulate diverse cellular functions including cell cycle arrest, apoptosis and autophagy. Silencing of Foxo1 resulted in a decrease in the conversion of LC3I to LC3II and inhibition of the autophagy in cardiomyocytes (23).

During oxidative stress in human chondrocytes, intracellular ROS and oxidized glutathione were increased, resulting in cell death**. **FOXO1 down-regulation significantly increased ROS-induced apoptosis with reduced level of ROS scavengers, such as GPX1 and catalase and decreased expression of autophagy related proteins (24). Akasaki and colleague, revealed that reduced apoptosis with decreased expression of proapoptotic mediators, caspases and TRAI was observed after FOXO1 silencing (25).

The Hippo-YAP- FOXO1 pathway also participates during I/R. The Hippo pathway negatively regulates cell growth and survival. YAP is the terminal effector of the Hippo pathway that interacts with FOXO1 in the cardiomyocyte nucleus. The consequence of Hippo activation was inactivation of YAP, suppression of FoxO1 activity with decreased catalase, and MnSOD antioxidant gene expression.

During ischemia/reperfusion in the heart, activation of Hippo led to down-regulation of antioxidant enzymes and promoted oxidative stress-induced cell death (26). I/R injury is a complex physiological process associated with the interaction of many genes and proteins that ultimately lead to liver failure (27).

Bcl-2 overexpression was reported in ischemic mouse livers after reperfusion that suppresses the release of cytochrome c and expression of caspase-3, protecting the liver against the ischemic injury by inhibiting apoptosis. Induction of apoptosis with activation of caspase 3 and Bax expression in the rat liver transplantation was confirmed during graft cold storage and after cold I/R (28).

In our study, we observed that the expression of miR-370 was up-regulated in hepatic I/R injury. This expression was accompanied with up-regulation of BAX and down-regulation of Bcl-2 gene. A reverse pattern was observed after BM-MSCs injection and hepatic ischemic preconditioning. It seems that MSCs are more effective, but both conditions activate signaling pathways that inhibit cell apoptosis and promote hepatocytes survival.

Preconditioning protects the hepatocytes against I/R damage by either increasing the expression of the autophagy-related protein LC3 and Beclin 1 and formation of autophagosomes and, on the other hand, by alleviating the apoptotic pathway (29, 30). Our protocol for ischemic preconditioning was effective only on miR-370 expression. Although the expression of Bcl2 and Bax genes was changed, it was not significant. In the current protocol, the liver underwent 10 min of hepatic ischaemia and 10 min of reperfusion prior to prolonged ischemic insult. Revising the protocol with more reperfusion time before prolonged ischemic insult may activate the protective pathways more efficiently and can protect the liver from subsequent major insult.

The MSCs have well known immunomodulatory, anti-inflammatory, and anti-apoptotic properties with paracrine or endocrine mechanisms that enhance the tissue repair. The exosome derived from MSCs protects the liver against hepatic I/R by decreasing the inflammatory response, alleviating the levels of apoptotic markers, like caspase-3 and bax, as well as increasing the levels of antioxidative markers, such as glutathione (GSH), glutathione peroxidase (GSH-Px) and superoxide dismutase (SOD) (31). Similar findings were confirmed in the studies that focused on protective role of these cells on hepatic I/R (7). 

Overall, these pathways are suggestive of the protective role of BM-MSCs against liver I/R:

• BM-MSCs down-regulate the TGF-β/ TβRII receptor signaling pathway which plays an essential role in liver injury and regeneration.

• BM-MSCs interact with Hippo/FoxO1 signaling pathway, up-regulate the antioxidant enzymes, and decrease the oxidative stress-induced cell death and apoptosis.

• On the other hand, BM-MSCs may increase the FOXO1 expression that ultimately increase the autophagy pathway that protects the hepatocytes.

• BM-MSCs might modulate hepatic lipid metabolism and ATP production which conserve the cell energy to maintain the integrity of the plasma membrane and mitochondria.

## Conclusion

 Our findings addressed the protective effect of BM-MSCs in hepatic I/R. Although the exact regulatory mechanism of miR-370 in the liver I/R has not been fully understood, BM-MSCs exerts its effect by reducing the expression of miR-370, BAX and up-regulating the Bcl2. More studies are required to further illuminate the probable mechanisms.
